# Evaluation of Inflammatory Markers as Prognostic Factors in the Treatment of Hepatocellular Carcinoma (HCC) with Degradable Starch Microspheres by Transarterial Chemoembolization (DSM-TACE)

**DOI:** 10.3390/cancers17040647

**Published:** 2025-02-14

**Authors:** Hannah L. Steinberg-Vorhoff, Andriana Tropotel, Jens M. Theysohn, Benedikt Schaarschmidt, Johannes Haubold, Matthias Jeschke, Leonie Jochheim, Johannes M. Ludwig

**Affiliations:** 1 Department of Diagnostic and Interventional Radiology and Neuroradiology, University Hospital Essen, University of Duisburg-Essen, Hufelandstr. 55, 45147 Essen, Germany; hannah.steinberg-vorhoff@uk-essen.de (H.L.S.-V.); jens.theysohn@uk-essen.de (J.M.T.); benedikt.schaarschmidt@uk-essen.de (B.S.); johannes.haubold@uk-essen.de (J.H.); 2 Institute of Diagnostic Radiology, Interventional Radiology and Nuclear Medicine, BG Clinics Bergmannsheil, Ruhr-University of Bochum, Buerkle-de-la-Camp Platz 1, 44789 Bochum, Germany; andriana.tropotel@bergmannsheil.de; 3 Department of Gastroenterology and Hepatology, University Hospital Essen, University of Duisburg-Essen, Hufelandstr. 55, 45147 Essen, Germany; matthias.jeschke@uk-essen.de (M.J.); leonie.jochheim@uk-essen.de (L.J.); 4 Department of Radiology and Nuclear Medicine, University Medical Center Mannheim, Heidelberg University, Theodor-Kutzer-Ufer 1-3, 68167 Mannheim, Germany

**Keywords:** carcinoma, hepatocellular, chemoembolization, therapeutic, degradable starch microsphere (DSM) TACE, inflammatory markers, bridging to transplantation

## Abstract

**Objective:** To evaluate the prognostic value of pre-therapeutic inflammatory markers before transarterial chemoembolization with degradable starch microspheres (DSM-TACE) in the treatment of hepatocellular carcinoma (HCC). **Methods:** A total of 155 patients (81% male, median age: 68 years) who underwent first-time DSM-TACE between 07/13 and 06/22 were included in the study. Inflammatory indices were dichotomized using median values. Cox proportional hazard model for univariate (UVA) and multivariate (MVA) analyses (hazard ratio; 95% CI, *p*-value) and Kaplan–Meier analyses (overall survival (OS) in months; 95% CI; log-rank test) were performed. **Results:** The median OS of the study cohort was 15.9 (12.9–20) months with a median survival according to BCLC stages A (12%), B (41%), and C (47%) of median not reached, 19.3 (15.3–27), and 7.2 (4.5–9.0) months, respectively (*p* < 0.0001). In the UVA, several inflammatory markers on OS were statistically significant with the systemic inflammatory response index (SIRI; ≤median (2.04) HR: 0.41 (0.19–0.89); *p* = 0.024) and the lymphocyte to monocyte ratio (LMR; >median (1.82) HR: 0.44 (0.2–0.9); *p* = 0.025) remaining statistically significant in MVA together with the BCLC stage (*p* = 0.0001), ALBI grade (*p* = 0.016), hepatic tumor burden (≤25% vs. >25%; *p* = 0.006), and largest HCC lesion (≤5.5 cm vs. >5.5 cm; *p* = 0.008). In subgroup analysis, patients with elevated LMR and reduced SIRI exhibited significantly prolonged overall survival (OS) in both BCLC B (*p* < 0.0001) and Child–Pugh A (*p* = 0.021) subgroups. **Conclusion:** The findings suggest that SIRI and LMR may serve as valuable tools in identifying BCLC B and Child–Pugh A patients who could potentially benefit better from DSM-TACE treatment. Nevertheless, further research is recommended to confirm these findings and to provide more comprehensive insights.

## Simple Summary

This retrospective study evaluated the prognostic significance of pretreatment inflammatory markers in patients with hepatocellular carcinoma (HCC) undergoing degradable starch microsphere (DSM)-TACE. The study findings, derived from data of 155 patients, indicated that two inflammatory markers, the systemic inflammatory response index (SIRI) and the lymphocyte-to-monocyte ratio (LMR), possessed significant prognostic value. Patients exhibiting lower SIRI or higher LMR had superior overall survival (OS), particularly those with intermediate cancer stages (BCLC stage B) and optimal liver function (Child–Pugh A). The median survival times exhibited notable variation across cancer stages, with early-stage patients demonstrating the most favorable outcomes. Multivariate analysis confirmed that, in addition to cancer stage, liver function (ALBI grade), tumor size, and liver tumor burden, these markers were independently associated with overall survival. These findings suggest that SIRI and LMR may serve as valuable tools in identifying BCLC B and Child–Pugh A patients who could potentially benefit better from treatment.

## 1. Introduction

Hepatocellular carcinoma (HCC) has a major impact on public health, as it is the fourth leading cause of cancer-related deaths worldwide, with incidence and cancer-specific mortality rates continuing to rise in many countries [1,2]. Unfortunately, most patients are diagnosed at an advanced stage, where resection and ablation can no longer be performed with curative intent. The European Association for the Study of the Liver recommends transarterial chemoembolization (TACE) as the first-line treatment option in their guidelines [3]. Among the various TACE techniques, degradable starch microsphere (DSM)-TACE, with its transient vessel occlusion approach, appears to be the gentlest TACE technique. It preserves liver function, is well tolerated by patients with acceptable adverse event rates, and demonstrates comparable treatment efficacy to conventional (c) and drug-eluting bead (DEB)-TACE, especially when treatment is conducted using a lobar or whole liver approach [4,5,6]. The Cardiovascular and Interventional Radiological Society of Europe (CIRSE) practical guidelines regard DSM-TACE as a particularly suitable treatment for patients with more advanced disease states (including patients with portal vein thrombosis), multifocal tumors, and suitable for patients with borderline preserved liver function [7].

Recent research has underscored the significance of systemic inflammation in patients with cancer, suggesting that it may influence disease progression and overall survival. The systemic inflammatory response to cancer is associated with alterations in various blood cells, including leukocytes and their subtypes, as well as platelets, and has been acknowledged as an indicator of the complex relationship between the tumor stromal microenvironment and immune response. Research indicates that inflammatory markers and indices derived from routinely assessed differential blood counts may serve as independent prognostic factors for various types of cancer, including HCC [8,9]. Although numerous studies have underscored the prognostic value of systemic inflammatory markers in c- and DEB-TACE, there is a dearth of published literature examining the prognostic significance of these markers in patients undergoing DSM-TACE [10,11]. This study aimed to retrospectively evaluate the prognostic potential of various systemic inflammatory markers in patients with hepatocellular carcinoma treated with DSM-TACE.

## 2. Materials and Methods

The study design and population included one hundred and fifty-five patients in this study. Patients received the first DSM-TACE treatment between 07/2013 and 06/2022, with censoring dated to 15 October 2023. The ethics committee granted approval, and the Institutional Review Board waived the need for informed consent. The interdisciplinary team opted for the use of DSM-TACE when surgery, ablation, conventional TACE, radioembolization, or systemic therapy previously failed or were not considered suitable or safe. Twenty patients in this study cohort have been previously reported [4,12]. Disease progression and possible therapies have been discussed by interdisciplinary tumor boards. Therapy was decided based on patient-specific factors, such as liver function, tumor size, extrahepatic spread, and patient status, as well as institutional experience.

Treatment and therapeutic concept: The DSM-TACE procedure was performed using EmboCept^®^ S particles (PharmaCept, Berlin, Germany) in an angiography suite, as previously described [12]. We conducted treatments on a “planned” and not on a “demand” basis. DSM-TACE was performed usually every 4–8 weeks and discontinued in the case of tumor progression or another treatment being performed (e.g., liver transplantation)

Inflammatory markers: The following inflammatory ratios and indices were calculated: Aggregate Index of Systemic Inflammation (AISI = (neutrophils × monocytes × platelets)/lymphocytes), c-reactive-protein to lymphocyte ratio (CRP/L = CRP/lymphocyte ratio), derived NLR (dNLR = neutrophils/(white blood cells—neutrophils), lymphocyte to monocyte ratio (LMR = lymphocytes/monocytes), neutrophil-to-lymphocyte ratio (NLR = neutrophil/lymphocytes), neutrophil-to-lymphocyte ratio (NLPR = neutrophil/(lymphocyte × platelet ratio)), platelet-to-lymphocyte ratio (PLR = Platelet/lymphocytes), systemic inflammatory index (SII = (neutrophils × platelets)/lymphocytes), and systemic inflammatory response index (SIRI = (neutrophils × monocytes)/lymphocytes). Cells were counted as numbers per nanoliter (/nL). Laboratory data were obtained before the first DSM-TACE.

Assessment of hepatic tumor response and survival: For one hundred and thirty-three (85.8%) patients, CT and MRI baseline and one follow-up imaging (median 4.7 days prior to the first treatment) were available as twenty-two died early due to tumor-related causes. Follow-up imaging was performed after every three treatment sessions or when tumor progression was suspected clinically. Treatment response was assessed according to the modified Response Evaluation Criteria in Solid Tumors (mRECIST) [13]. TTP was calculated from the date of the first DSM-TACE procedure. In case of loss to follow-up imaging, the date of the last imaging was used as the censoring date.

Statistics: The median OS and time to progression (TTP) were calculated using Kaplan–Meier analysis. For univariate (UVA) and multivariate (MVA) analyses, Cox proportional hazard model analysis was applied to calculate hazard ratios (HRs) and 95% confidence intervals (CIs). Contingency analysis was performed using the Pearson’s correlation coefficient. Indices and ratios were dichotomized using the median, whereas others were dichotomized using the upper level of normal (GGT, LDH) or at 20 ng/mL for AFP. Statistical analyses were performed using JMP 17.0 (SAS Institute Inc., Cary, NC, USA). Statistical significance was set at *p* < 0.05.

## 3. Results

### 3.1. Demographics

One hundred and fifty-five patients (81% men; 93% Caucasians) with a median age of 68 years (range: 46–87 years) were included in this study. The initial treatment was performed between July 2013 and June 2022, and the censoring date for follow-up was 15 October 2023. In total, 46 patients were lost to follow-up, with a median follow-up time of 26.4 months (95% CI: 19.4–30 months). The additional baseline characteristics of the study cohort are shown in Table 1.

### 3.2. Treatment Characteristics

A total of 580 treatments were performed, with a median of three (range: 1–13) treatments per patient. Treatment was most commonly performed via the bilobar/residual liver after hemihepatectomy (50%), followed by lobar (40.5%), and (bi)segmental/selective (9.5%) embolization. A median of 450 mg (range: 68–980 mg) of EmboCept^®^ S particles was mixed with doxorubicin in 97.8% of cases (median: 50 mg) and only 2.2% with Mitomycin C (median of 5 mg).

### 3.3. Survival Analysis

**Figure 1:**
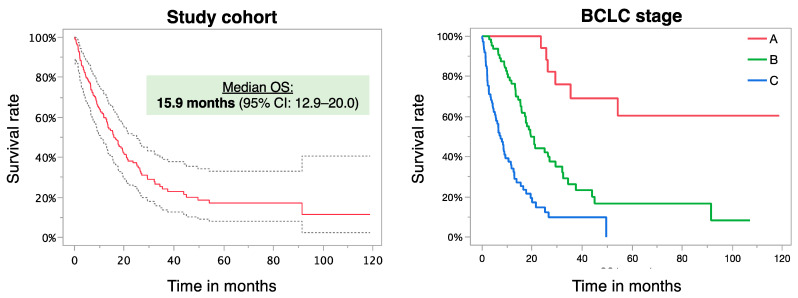
Overall survival of all patients (**left**) and overall survival according to BCLC stage (**right**) following the first DSM treatment.

The median survival time of the study cohort was 15.9 months (95% CI: 12.9–20) with 6-month, 1-year, 2-year, and 3-year survival rates of 75.3%, 55.8%, 29.2%, and 13.0%, respectively. The median OS times according to BCLC stage A/B/C were as follows: median not reached, 19.3 (95% CI: 15.3–27), and 7.2 (95% CI: 4.5–9.0) months, respectively (*p* = 0.001) (Figure 1). The median OS times stratified for the Child–Pugh class A/B/C were 23.5 (95% CI: 17.1–32.1), 10.4 (95% CI: 6.5–15.5), and 5.2 (1.4–.) months, respectively (*p* < 0.0001). In UVA, several pretreatment factors were found to be significantly associated with OS (Table 2).

In addition to significantly longer OS for patients with lower BCLC stage and Child–Pugh class, a survival benefit was also observed for lower ALBI grades, with a median of 26.3, 14.5, and 9.2 months for grades 1–3, respectively (*p* = 0.014). Furthermore, patients with no or slight ascites with a hepatic tumor burden of up to 25% and a maximum tumor size of the largest lesion of up to 5.5 cm as well as patients without vascular invasion, survived significantly longer.

SII, NLR, dNLR, LMR, CRP, SIRI, and AISI were identified as significant inflammatory markers, whereas NLPR, PLR, and CRP/L were not. Patients with Lactate Dehydrogenase (LDH) and Gamma-glutamyl Transpeptidase (GGT) levels within the normal range also survived significantly longer. Notably, the elevation of other liver enzymes was not a statistically significant OS factor.

**Figure 2:**
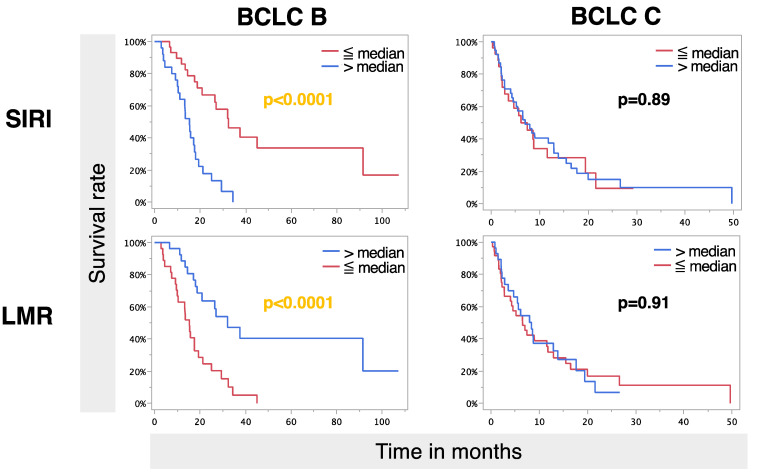
Overall survival rates of SIRI and LMR according to BCLC stages B and C following the first DSM-TACE treatment.

In the MVA group, several factors remained independent predictive factors for OS, with BCLC remaining the strongest factor (*p* = 0.0001). Furthermore, the ALBI grade, tumor burden, and size of the largest liver lesion remained significant. Regarding inflammatory markers, only LMR and SIRI remained significant. Further subanalysis revealed that a survival benefit for both higher LMR and lower SIRI was seen in BCLC B patients with a longer median OS of 32.0 months (95% CI: 18.7–.) vs. 15.3 (95% CI: 9.7–19.3) for higher LMR and a prolonged median OS of 32.4 months (95% CI: 20.9–91.5) vs. 15.3 (95% CI: 10.3–27.0) for lower SIRI (*p* < 0.0001). No significant difference was observed in BCLC stage C (Figure 2).

Similar findings were observed for the Child–Pugh score, with longer survival times for higher LMR with 26.3 months (95% CI: 17.1–.) vs. 19.3 months (95% CI: 13.0–29.3) (*p* = 0.021) and for lower SIRI with 54.2 months (95% CI: 26.3–.) vs. 13.9 months (95% CI: 9.0–18.0) (*p* < 0.0001) in Child–Pugh A patients. No statistical difference was seen for Child–Pugh class B and C. Notably, the proportions of high SIRI and low LMR increased in higher Child–Pugh classes (only significant for LMR, *p* = 0.0013, with low LMR proportions of 38.4%, 62%, and 84.6% for Child–Pugh class A/B/C, respectively).

### 3.4. Response Analysis

**Figure 3:**
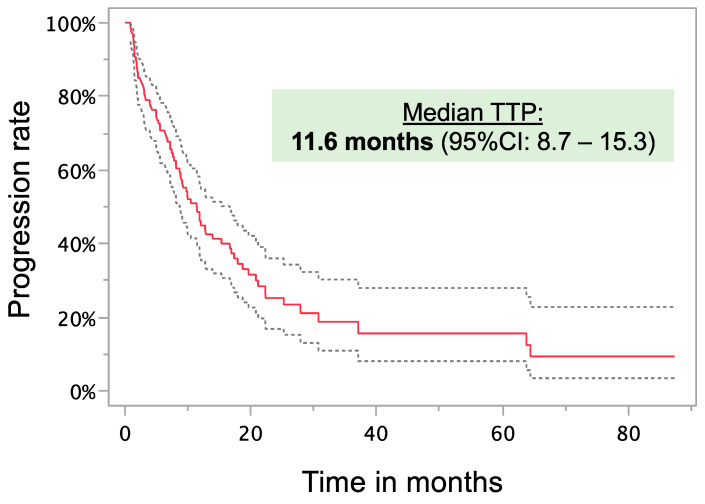
Time to progression (TTP) in all patients following the first DSM treatment.

The median TTP was 11.6 months (95% CI: 8.7–15.3) (Figure 3). The results from the best response according to mRECIST with median survival and TTP for each group are shown in Table 3. It should be noted that imaging was not routinely performed after each imaging, which may have biased the analysis. Regarding best achieved response and inflammation, the rate of patients with SIRI > median was the highest for PD (progressive disease, 79.0%), followed by PR (partial response, 51.4%), SD (stable disease, 43.8%), and CR (complete response, 26.7%) (*p* = 0.015). Statistical significance was also seen for LMR > median (CR: 73.3%; PR: 45.7%; SD: 58.3%; PD: 21.1%; *p* = 0.011), dNLR > median (CR: 26.7%; PR: 42.3%; SD: 55.3%; PD: 73.7%; *p* = 0.033), and non-elevated CRP (CR: 58.8%; PR: 42.9%; SD: 26.4%; PD: 17.4%; *p* = 0.016). All significant factors in the MVA of OS were included in the TTP UVA. Only BCLC stage, hepatic tumor burden, size of the largest lesion, and SIRI were significant factors in the univariate analysis. In the MVA group, only BCLC stage and hepatic tumor burden remained independently significant.

DSM-TACE for bridging to liver transplantation: Fifteen patients were listed for transplantation, representing 9.7% of the study cohort. In one patient, downstaging was observed following DSM-TACE; thus, the patient was listed consecutively following a single treatment session with DSM-TACE. The patient also received cTACE as a bridging therapy following the listing. Another patient was treated with DSM-TACE but was only listed after thermal ablation and radioembolization therapy as the bridging modality.

In the remaining 13 cases, DSM-TACE was selected as the primary bridging therapy in accordance with the recommendations of the interdisciplinary tumor board, as radioembolization or other locoregional therapies were deemed inferior or unsafe options. The median MELD Score was 12 (range: 7–20). Two patients were unlisted because of tumor progression after 3.9 and 4.3 months, respectively. Another patient was unlisted due to a lowered MELD Score of 6 and was lost to follow-up 26.5 months later without observed tumor progression after the initial partial response.

A total of ten patients were actually transplanted after a median of 6.9 months, ranging from 3.1 to 16.3 months following the first DSM-TACE session. In total, patients received a median of three (range 1–6) treatment sessions when transplanted, compared to two (range 1–7) treatments when unlisted.

Following transplantation, two patients developed extrahepatic metastases, with one in the bone 16 months after transplantation and the other patient developed lung metastases 6 months after transplantation. Both patients had G3 tumors, and the latter also had histologically proven lymph node metastases in the liver hilum. Notably, tumor progression following transplantation was not observed in patients with G1 or G2 tumors. Two additional patients died early after transplantation due to early portal vein thrombosis with bleeding complications and liver failure, as well as pneumogenic sepsis (*S. aureus*) in another patient. In the remaining six patients, no death or tumor recurrence were observed during a median follow-up time of 51.3 months (range: 3.6–107.3 months).

The tumor necrosis rate was recorded in seven patients, with a total of nine lesions reported. The median tumor necrosis rate was 95% (range: 5–100%). The mean necrosis rate was 68.9%. In total, a 100% tumor necrosis rate was observed in four lesions (44%).

## 4. Discussion

The results of this retrospective study demonstrated that transarterial chemoembolization with degradable starch microspheres is an effective treatment option when other treatment approaches have failed or are not viable due to perceived elevated risks. In general, the median survival rates of 15.5 months are in line with a recent review (range 11.3–36 months) [5].

In addition, to the well-established risk factors for OS and TTP, numerous systemic inflammation ratios and indices have been identified as prognostic factors associated with OS and TTP.

It is well established that inflammation plays a pivotal role in the development of HCC and is a crucial factor in stimulating the proliferation and dedifferentiation of tumor cells, as well as in promoting evasion from the anticancer immune response [5]. Research has shown that peripheral blood cells, such as lymphocytes, monocytes, neutrophils, and platelets, play a pivotal role in modulating tumor growth, invasion, metastasis, and anticancer immune responses [14]. Despite the intricate interplay of immune cells within the tumor microenvironment, with varying pro- and anticancer functions exhibited by different immune cells and phenotypes, an overall pro- or anticancer tendency can be attributed to distinct immune cells. In general, lymphocytes are crucial for immunosurveillance and immune editing. Their infiltration into the tumor microenvironment enhances the immune response against cancer. Furthermore, the presence of tumor-infiltrating lymphocytes is also linked to better survival rates, whereas low lymphocyte levels and inadequate tumor infiltration are associated with poorer outcomes [15]. Conversely, Tumor-associated macrophages (TAM) are a specific subset of monocytes that originate from the bloodstream and are activated in response to tumor-released chemokines. These macrophages secrete growth factors, cytokines, and pro-angiogenic factors, which can directly and indirectly affect tumor cells by altering the tumor microenvironment. This can promote tumor growth, stimulate invasive migratory ability, and enhance cell stemness, treatment resistance, and treatment resistance. Additionally, numerous studies have validated the link between high levels of TAM infiltration and poor prognosis [16,17].

Neutrophils play a critical role in the development of cancer by promoting carcinogenesis. They contribute to cancer initiation by activating inflammatory pathways, causing DNA damage through the release of genotoxic substances, and supporting tumor progression by facilitating neoangiogenesis and suppressing the immune responses [18]. Elevated platelet levels have been linked to inferior OS and a heightened incidence of extrahepatic metastases [19,20]. Platelets facilitate tumor progression by sustaining cancer stem cell survival, stimulating angiogenesis, enhancing cell proliferation, and aiding immune evasion [21].

A number of ratios and indices derived from peripheral differential blood counts have been proposed as viable, accessible, cost-effective, and reliable methodologies for developing prognostic scores [10]. In this study, a high LMR (>1.82) and low SIRI (≤2.04) were identified as independent prognostic factors for OS, demonstrating a median OS of approximately twice as long. The observed phenomenon can be attributed to the relative abundance of anticancer leukocytes compared to monocytes in the lymphocyte-to-monocyte ratio and monocytes and neutrophils in the systemic inflammatory response index. This suggests that a high SIRI and low LMR reflect a suppressed immune response, leading to poorer outcomes in HCC patients [22,23]. TACE has previously been shown to reduce the density of immune-exhausted effector cytotoxic T-cells and regulatory T-cells (Tregs) within the tumor, while significantly upregulating pro-inflammatory pathways [24]. This suggests that TACE can alter the tumor microenvironment to become more immunogenic. Additionally, TACE has been associated with increased infiltration of immune cells, such as CD3, CD4, and CD8 T-lymphocytes into the tumor, as well as higher expression of immune checkpoint markers, such as PD-1 and PD-L1 [25,26]. Overall, it may be hypothesized that the interplay of a rather antitumor immune system constellation, as indicated by elevated LMR and low SIRI, may allow for a subsequent antitumor response following TACE, which may affect the outcome, in addition to the direct antitumor effects induced by the TACE procedure. Yet, this remains to be investigated.

A number of studies on c- and DEB-TACE have investigated LMR as a prognostic marker, utilizing cut-offs of 2.2 and 2.24, respectively [10,11,27]. It is imperative to acknowledge that Liu et al. included BCLC B and C patients with only large HCC tumors, with an average size of 12.1 cm, which was significantly larger than that in our cohort, while the median tumor size of 4.5 cm in the study by Minici et al. was comparable to that in our cohort, and only BCLC B patients were included. Although Liu et al. included BCLC B and C patients, they did not perform subgroup analysis; thus, it remains unclear whether the observed prognostic value is limited to BCLC B patients, as in our cohort. Additionally, both employed a receiver operating characteristic (ROC) analysis to determine cut-off values, utilizing a dichotomized approach based on 3-month tumor progression status and 6-month progression-free status, restricting the ability to directly compare and transfer cut-off values [11,27]. Regarding SIRI, a comparable survival benefit with an almost doubled rate (1046 days for low (≤0.88) and 425 days for high (>0.88) SIRI) was identified by Wang et al. in patients treated with conventional and DEB-TACE [28]. However, it is important to note that 64.4% of the patients were classified as BCLC A, which is different from the 11% observed in the current group. Furthermore, no subgroup analysis was conducted to examine the survival outcomes of SIRI in patients categorized according to the BCLC system. This limits our ability to compare our findings with those of other studies. Notwithstanding the evident disparities observed in the study population, the utilization of disparate TACE techniques, and the analysis conducted, the overarching pattern and the significance of LMR and SIRI remain consistent, thereby underscoring its importance in patients undergoing TACE treatment.

Concerning DSM-TACE, there is a paucity of published data on the potential role of systemic inflammatory markers. Minici et al. observed that patients with intermediate-stage HCC who underwent successful downstaging to transplantation exhibited lower NLR (≤7.2) and LMR (>4) [29]. Although downstaging was not the focus of this study, statistically significant differences were observed in the proportions of patients with high versus low LMR, SIRI, dNLR, and elevated versus non-elevated CRP levels regarding the best achieved response. In accordance with our findings, a multicenter study on conventional and DEB-TACE revealed a prolonged progression-free survival for high LMR, and a single-center study on conventional TACE also identified low SIRI values as a prognostic factor for longer progression-free survival and prolonged OS [28,30]. Furthermore, SIRI has been associated with higher tumor aggressiveness, which often presents a greater challenge in terms of treatment effectiveness [31].

In general, the selected cut-off values and utilized methodologies (receiver operating curves vs. median vs. lack of clear documentation) exhibit significant variability across various research publications. This questions whether a standardized and reliable cut-off can be identified, or if cut-off values should be tailored to each treatment population at each institution to be used as a prognostic factor for treatment decision-making.

In this study of thirteen patients listed for transplantation, only four (31%) were classified as BCLC A, while seven (54%) and two (15%) were classified as BCLC B and C, respectively. In a study conducted by Minici et al. on bridging with DSM-TACE, only patients with BCLC stage A were included [32]. Moreover, the median serum bilirubin in this study was higher, at 1.95 mg/dL, compared to 1.0 mg/dL. These differences underscore the more advanced stage and impaired liver function of patients in our study. Nevertheless, 76.9% of patients underwent transplantation, in contrast to the 33% observed in the study by Minici et al. It is noteworthy that the waiting period for transplantation was shorter in our study, with a median of 6.9 months, compared with 11.9 months. This may have contributed to the lower dropout rate. An increase in VEGF plasma levels following TACE has been identified as a negative prognostic indicator, correlating with poorer tumor response rates and reduced progression-free survival [33]. This elevation is linked to a greater likelihood of local tumor recurrence and the spread of distant metastases. Additionally, elevated VEGF may contribute to more aggressive HCC characteristics, such as infiltrative or metastatic transformations, and the formation of collateral tumor blood supplies, which can lead to resistance against TACE treatment [34]. The administration of DSM-TACE results in a comparatively diminished augmentation of vascular endothelial growth factor (VEGF) in the serum when contrasted with cTACE [35]. Overall, DSM-TACE may serve as a viable bridging method to transplantation, especially when other locoregional therapies are not feasible.

This study has several limitations, including its retrospective nature and the use of data from only one institution. Furthermore, the cut-offs determined in this study warrant verification with a control cohort, ideally from another institution. Additionally, the study cohort was heterogeneous, as is typical in real-world scenarios. This characteristic may introduce confounding variables that could potentially affect the findings of this study. Therapy decisions were made via an interdisciplinary tumor board. In another institute, a different therapy might have been chosen for the patient, and a different patient cohort could have shown different results; therefore, selection bias is an important limitation in our study. Further multicenter studies are necessary to validate these results.

## 5. Conclusions

These findings suggest that the systemic inflammation markers SIRI and LMR may serve as valuable tools in identifying BCLC B and Child–Pugh A patients who could potentially benefit better from DSM-TACE treatment. Nevertheless, further research is recommended to confirm these findings and to provide more comprehensive insights.

## Figures and Tables

**Table 1:** Patients’ baseline characteristics.

Baseline Characteristic	Number of Patients
Cirrhosis	153 (98.7%)
Etiology of cirrhosis	
• Alcohol	33 (21.3%)
• Non-alcoholic steatohepatitis	33 (21.3%)
• Viral	37 (23.9%)
• Mixed	15 (9.7%)
• Other	7 (4.5%)
• Unknown	28 (18.1%)
Ascites	
• None	73 (47.1%)
• Mild	64 (41.3%)
• Moderate to severe	18 (11.6%)
Disease extent	
• Bilobar	111 (71.6%)
• Unilobar	44 (28.4%)
Number of lesions	
• Uninodular	28 (18.1%)
• 2–3 nodules	43 (27.7%)
• Multinodular (>3 nodules)	84 (54.2%)
Largest Lesion, median (range)	5.5 cm (0.8–11.0 cm)
Presence of portal vein invasion	43 (27.7%)
Extrahepatic metastases present	41 (26.5%)
Child–Pugh class	
• A	84 (54.2%)
• B	59 (38.1)
• C	12 (7.7%)
BCLC stage	
• A	17 (11.0%)
• B	64 (41.3%)
• C	74 (47.7%)
ECOG	
• 0	83 (53.5%)
• 1	57 (36.8%)
• 2	11 (5.8%)
• 3	2 (7.1%)
• Unknown	2 (7.1%)
Pretreatment	95 (61.3%)
• Resection	15 (9.7%)
• Ablation	11 (7.1%)
• cTACE	38 (24.5%)
• Bland Embolization	1 (0.65%)
• Radioembolization	61 (39.4%)
• Sorafenib	9 (5.8%)
• Lenvantinib	3 (1.9%)
• Liver transplantation	1 (0.65%)
• Radiation therapy	1 (0.65%)

Patients’ baseline characteristics. Patients may have received more than one prior therapy. Abbreviations: BCLC (Barcelona Clinic Liver Cancer); ECOG (Eastern Cooperative Oncology Group); cTACE (conventional transarterial chemoembolization). Percentages shown reflect the percentage of the entire study cohort. The median size of the largest liver lesion is stated in cm incl. the range.

**Table 2:** Uni- and multivariate survival analysis of pretreatment factors.

			**Univariate Analysis**		**Multivariate Analysis**	
Subgroups		Median OS in months (95% CI)	HR (95% CI)	*p*-value	HR (95% CI)	*p*-value
Gender	Female	17.1 (6.2–29.3)	0.97 (0.6–1.55)	0.9	—	—
	Male	15.8 (12.7–20.0)	1		—	
Age (in years)	≥70	14.5 (9.4–18.0)	1.43 (0.97–2.1)	0.07	—	—
	<70	18.7 (13.2–26.5)	1		—	
Child–Pugh class *	A	23.5 (17.1–32.1)	0.33 (0.16–0.68)	**<0.0001**	0.49 (0.13–1.9)	0.39
	B	10.4 (6.5–15.5)	0.8 (0.39–1.65)		0.46 (0.15–1.4)	
	C	5.2 (1.4–.)	1		1	
BCLC stage **	A	Not reached (29.3–.)	0.09 (0.04–0.2)	**<0.0001**	0.12 (0.04–3.7)	**0.0001**
	B	19.6 (15.9–19.3)	0.34 (0.2–0.5)		0.35 (0.19–0.65)	
	C	7.5 (4.7–10.6)	1		1	
ALBI grade ***	1	26.3 (17.7–49.7)	0.44 (0.25–0.76)	**0.014**	0.22 (0.08–0.64)	**0.016**
	2	14.5 (10.6–20.0)	0.66 (0.42–1.05)		0.39 (0.18–0.84)	
	3	9.2 (3.6–15.5)	1		1	
Ascites ****	No	23.6 (18.0–32.4)	0.22 (0.12–0.4)	**0.0001**	0.28 (0.1–0.8)	0.058
	slight	11.8 (8.0–17.5)	0.53 (0.35–0.8)		0.4 (0.17–0.93)	
	moderate/severe	5.2 (2.1–13.4)	1		—	
Hepatic tumor burden	≤25%	20.9 (15.8–26.5)	0.28 (0.18–0.44)	**<0.0001**	0.33 (0.15–0.72)	**0.006**
	>25%	6.6 (2.8–11.1)	1		1	
Number of HCC lesions *****	1	25.0 (13.0–29.4)	0.58 (0.34–0.98	0.055	—	—
	2–3	20.8 (8.9–54.2)	0.67 (0.43–1.03)		—	
	>3	15.5 (8.6–17.8)	1		—	
Largest HCC lesion	≤median (5.5 cm)	25.2 (16.5–32.4)	0.44 (0.3–0.64)	**<0.0001**	0.4 (0.2–0.79)	**0.008**
	>median	10.1 (7.5–15.3)	1		1	
Vascular invasion	No	20.9 (15.9–26.5)	0.29 (0.18–0.46)	**<0.0001**	0.98 (0.47–2.06	0.97
	Yes	8.0 (4.5–10.6)	1		1	
SII	≤median (501.2)	19.5 (9.7–29.4)	0.66 (0.44–0.99)	**0.048**	0.77 (0.32–1.9)	0.55
	>median	15.3 (11.6–18.0)	1		1	
NLR	≤median (3.46)	19.5 (11.8–32.1)	0.61 (0.4–0.93)	**0.02**	0.4 (0.15–1.04)	0.06
	>median	15.3 (10.1–17.7)	1		1	
dNLR	≤median (1.72)	18.7 (14.5–29.4)	0.6 (0.4–0.91)	**0.015**	0.74 (0.42–1.3)	0.31
	>median	16.5 (8.8–17.7)	1		1	
NLPR	≤median (1.72)	17.5 (9.0–25.0	0.91 (0.61–1.37)	0.65	—	—
	>median	15.4 (11.8–21.3)	1		—	
LMR	>median (1.82)	23.6 (14.5–37.5)	0.44 (0.29–0.67)	**<0.0001**	0.44 (0.2–0.9)	**0.025**
	≤median	11.8 (7.5–15.8)	1		1	
PLR	≤median (130.1)	19.3 (11.8–26.7)	0.69 (0.46–1.04)	0.075	—	—
	>median	14.5 (10.1–17.7)	1		—	
CRP	Not elevated	29.3 (19.5–44.0)	0.42 (0.27–0.65)	**<0.0001**	0.86 (0.47–1.58)	0.62
	elevated	12.7 (8.4–15.5)	1		1	
CRP/L	≤median (0.308)	13.4 (7.5–17.1)	0.92 (0.57–1.49)	0.73	—	—
	>median	8.8 (4.5–15.5)	1		—	
SIRI	>median (2.04)		1	**<0.0001**	1	**0.024**
	≤median	26.5 (17.5–45.0)	0.39 (0.25–0.6)		0.41 (0.19–0.89)	
AISI	≤median (291.3)	21.6 (11.8–32.4)	0.59 (0.4–0.89)	**0.012**	0.78 (0.3–1.19)	0.59
	>median	14.5 (10.1–17.7)	1		1	
AFP	≤20 ng/ml	25.7 (16.5–34.4)	0.51 (0.33–0.77)	**0.001**	0.83 (0.71–2.03)	0.49
	>20 ng/ml	13.4 (8.0–17.1)	1		1	
LDH	≤247 U/L	20.9 (15.4–32.4)	0.55 (0.38–0.8)	**0.002**	0.47 (0.26–0.86)	0.013
	>247 U/L	9.2 (7.1–16.5)	1		1	
GGT	≤55 U/L	35.4 (16.5–.)	0.47 (0.26–0.86)	**0.014**	0.66 (0.31–1.37)	0.26
	>55 U/L	14.5 (10.6–19.3)	1		1	
Prior therapy	No	12.9 (8.0–18.7)	1.3 (0.89–1.9)	0.19	—	—
	Yes	17.6 (13.5–21.6)	1		—	
Extrahepatic metastases	No	17.6 (13.4–20.9)	0.94 (0.61–1.45)	0.77	—	**—**
	Yes	10.4 (6.2–35.4)	1		—	

Uni- and multivariate survival analysis of pretreatment factors. AFP, Alpha-Fetoprotein; AISI, Aggregate Index of Systemic Inflammation; ALBI, Albumin-Bilirubin; BCLC, Barcelona Clinic Liver Cancer; CRP, c-reactive protein; CRP/L, CRP to leukocyte ratio; dNLR, derived neutrophil to lymphocyte ratio; GGT, Gamma-glutamyl Transpeptidase; LDH, Lactate Dehydrogenase; LMR, lymphocyte to monocyte ratio; NLPR, neutrophil to lymphocyte, platelet ratio; NLR, neutrophil to lymphocyte ratio; PLR, platelet to lymphocyte ratio; SII, Systemic Inflammatory Index; SIRI, Systemic Inflammation Response Index. * Child–Pugh class: Only the differences between A and B (*p* < 0.0001) and A vs. C (*p* = 0.0027) were statistically significant in the subgroup analysis of UVA. ** BCLC stage: In the subgroup analysis, all groups were statistically significant different, with a *p*-value ≤ 0.0018. *** Only ALBI grades 1 and 3 were statistically significantly different, with a *p*-value of 0.004. **** Differences between groups were significant, with a *p*-value ≤ 0.003. ***** Difference between 1 and >3 tumors was statistically significant (*p* = 0.044).

**Table 3:** Best achieved response.

Best Response	%	Median Number of Treatments (Range) Until Best Response	Median Time to Best Response (95% CI) in Months *	Median OS (95% CI) in Months *	Median TTP (95% CI) in Months *
CR	12.1%	3 (1–13)	7.7 (3.6–13.7)	91.5 (27.0–.)	27.9 (11.4–.)
PR	31.8%	2 (1–7)	3.5 (1.8–4.2)	21.3 (17.7–32.4)	15.3 (9.7–20.8)
SD	39.4%	1 (1–3)	1.5 (1.4–1.7)	13.9 (10.4–21.6)	9.3 (7.2–16.5)
PD	16.7%	1 (1–3)	1.8 (1.6–2.5)	6.5 (3.6–12.7)	1.7 (1.3–2.5)

Best response analysis data are shown with median overall survival (OS) and median time to progression (TTP). * The differences in median time to best response, overall survival, and time to progression were statistically significant (*p* < 0.0001). CR, complete response; PR, partial response; SD, stable disease; PD, progressive disease.

## Data Availability

The data that support the findings of this study will be made available upon reasonable request, subject to approval by the institutional Ethics Committee. To protect participant privacy, the data will be anonymized and stripped of all personally identifiable information before sharing. Interested researchers should submit a formal request detailing the intended use of the data. If approved, data access will be granted under a strict data use agreement that outlines terms for data security, confidentiality, and limitations on data reuse or redistribution. The time frame for data availability will be specified upon approval, typically within 6–12 months after publication. For inquiries about data access, please contact provide appropriate contact information or process.
